# Fever Incidence Is Much Lower in the Morning than the Evening: Boston and US National Triage Data

**DOI:** 10.5811/westjem.2020.3.45215

**Published:** 2020-06-24

**Authors:** Charles Harding, Francesco Pompei, Samantha F. Bordonaro, Daniel C. McGillicuddy, Dmitriy Burmistrov, Leon D. Sanchez

**Affiliations:** * Independent Statistical Analyst, Seattle, Washington; † Exergen Corporation, Watertown, Massachusetts; ‡ Gates Vascular Institute, Professional Emergency Services, Buffalo, New York; § Saint Joseph Mercy Hospital, Department of Emergency Medicine, Ann Arbor, Michigan; ¶ University of Michigan, Department of Emergency Medicine, Ann Arbor, Michigan; || Independent Statistical Analyst, Massachusetts; # Beth Israel Deaconess Medical Center, Department of Emergency Medicine, Boston, Massachusetts; ** Harvard Medical School, Boston, Massachusetts

## Abstract

**Introduction:**

In this observational study, we evaluated time-of-day variation in the incidence of fever that is seen at triage. The observed incidence of fever could change greatly over the day because body temperatures generally rise and fall in a daily cycle, yet fever is identified using a temperature threshold that is unchanging, such as ≥38.0° Celsius (C) (≥100.4° Fahrenheit [F]).

**Methods:**

We analyzed 93,225 triage temperature measurements from a Boston emergency department (ED) (2009–2012) and 264,617 triage temperature measurements from the National Hospital Ambulatory Medical Care Survey (NHAMCS, 2002–2010), making this the largest study of body temperature since the mid-1800s. Boston data were investigated exploratorily, while NHAMCS was used to corroborate Boston findings and check whether they generalized. NHAMCS results are nationally representative of United States EDs. Analyses focused on adults.

**Results:**

In the Boston ED, the proportion of patients with triage temperatures in the fever range (≥38.0°C, ≥100.4°F) increased 2.5-fold from morning to evening (7:00–8:59 PM vs 7:00–8:59 AM: risk ratio [RR] 2.5, 95% confidence interval [CI], 2.0–3.3). Similar time-of-day changes were observed when investigating alternative definitions of fever: temperatures ≥39.0°C (≥102.2°F) and ≥40.0°C (≥104.0°F) increased 2.4- and 3.6-fold from morning to evening (7:00–8:59 PM vs 7:00–8:59 AM: RRs [95% CIs] 2.4 [1.5–4.3] and 3.6 [1.5–17.7], respectively). Analyses of adult NHAMCS patients provided confirmation, showing mostly similar increases for the same fever definitions and times of day (RRs [95% CIs] 1.8 [1.6–2.1], 1.9 [1.4–2.5], and 2.8 [0.8–9.3], respectively), including after adjusting for 12 potential confounders using multivariable regression (adjusted RRs [95% CIs] 1.8 [1.5–2.1], 1.8 [1.3–2.4], and 2.7 [0.8–9.2], respectively), in age-group analyses (18–64 vs 65+ years), and in several sensitivity analyses. The patterns observed for fever mirror the circadian rhythm of body temperature, which reaches its highest and lowest points at similar times.

**Conclusion:**

Fever incidence is lower at morning triages than at evening triages. High fevers are especially rare at morning triage and may warrant special consideration for this reason. Studies should examine whether fever-causing diseases are missed or underappreciated during mornings, especially for sepsis cases and during screenings for infectious disease outbreaks. The daily cycling of fever incidence may result from the circadian rhythm.

## INTRODUCTION

As part of the circadian rhythm, body temperature generally rises and falls in a daily cycle, reaching its lowest values in the morning and its highest values in the afternoon and evening. The daily cycle of body temperature is a well-established aspect of human physiology that has links to the body’s clock, sleep patterns, metabolism, and other bodily functions.^1^ It is observed in both health and disease, although its form is modified by some diseases.^2^,^3^ For example, febrile diseases often produce exaggerated versions of the daily cycle of body temperature, in which normal or somewhat elevated temperatures occur in the morning and especially heightened temperatures occur in the afternoon and evening. Although other patterns of body temperature are also observed in febrile disease, this is the most common pattern.^2^–^4^

Despite the daily cycle of body temperature, fever is identified using a constant temperature threshold, such as ≥38.0° Celsius (C) (≥100.4° Fahrenheit [F]). It has been suggested that using a constant threshold to identify fever could lead to misdiagnosis because of the daily cycling of body temperature.^5^–^8^ In particular, fever-causing illnesses might be missed or underestimated in patients who present during the morning, since body temperature is usually lowest at that time.^5^–^9^ Additionally, fever false-positives could occur during the late afternoon and evening, when nonfebrile individuals generally have their highest body temperatures.^1^,^5^,^6^

The idea that common definitions of fever are inconsistent with the cycle of body temperature was discussed almost 150 years ago by one of the founders of medical thermometry, Carl Wunderlich.^6^ Since then, studies have found that in-patients at high risk of fever are least likely to reach the fever range in the morning,^7^,^8^,^10^ that healthy temperature percentiles are lowest in the morning,^5^ and that use of endotoxin to induce fever in healthy men produces lower temperature rises during mornings than evenings.^11^ Although these studies contribute useful evidence, they were limited to select patient groups^10^ and unusual experimental settings,^11^ or simply included too few febrile patients (n<40^5^,^7^,^8^) to determine whether the time-of-day changes in fever incidence were small or large. Consequently, it is still unclear whether the daily cycles of fever incidence are common and large enough to be clinically relevant—or whether they are specific to nongeneralizable settings, or are simply too small to be of any practical relevance at all.

Here, our primary aim was to estimate the time-of-day variations in the incidence of fever that is observed at emergency department (ED) triage, including nationally generalizable results. Additionally, we performed several secondary analyses to examine the relationship between fever incidence and the circadian rhythm, including multivariable regression analyses that were used to adjust for potential confounders, evaluations of diurnal changes in temperature means and standard deviations that were used to relate the incidence of fever to more typical body temperatures, comparisons of younger and older adults that were used to examine age-associated body temperature changes (such as the “older is colder” phenomenon),^12^,^13^ and comparisons of weekdays and weekends that were used to check for the effects of weekly schedules.

**Population Health Research Capsule**

What do we already know about this issue?

*Fever is identified with a fixed cutoff, such as ≥38.0° Celsius (≥100.4° Fahrenheit), yet body temperature usually changes from a morning low to an evening high*.

What was the research question?

How does the observed incidence of fever change over the day? Could morning cases be missed by the cutoff?

What was the major finding of the study?

*Fever-range temperatures were observed about half as often during mornings as during evenings at adult triages*.

How does this improve population health?

*Fever cutoffs may often miss fever-causing diseases in the morning. This should be considered in morning case management and during infectious disease screens*.

## METHODS

### Study Planning

This observational study used data from two sources: a Boston ED and a nationally representative survey of United States (US) EDs.^14^,^15^ The Boston data were initially collected to assess fever incidence as a means of tracking disease outbreaks (syndromic surveillance).^16^ We also observed that mean body temperature followed a consistent diurnal cycle across days of the week and seasons of the year in the Boston ED.^17^ These results suggested that it would be useful to study how body temperature cycles relate to fever presentation. However, except for Wunderlich’s research from the 1800s,^6^ little evidence was available to prepare specific research hypotheses. Therefore, we analyzed the Boston data exploratorily. Having done so, we used the national data to determine whether Boston results could be corroborated in an independent dataset, and to examine whether they generalized.

### Settings and Participants

The Boston study was conducted at the ED of Beth Israel Deaconess Medical Center (Boston, MA) from September 2009–March 2012. During this period, 115,149 temperatures were collected with data-logging thermometer systems during initial triage vital signs assessments. The national data were collected as part of the National Hospital Ambulatory Medical Care Surveys (NHAMCS) of the US Centers for Disease Control and Prevention. NHAMCS includes a nationally representative, multi-stage probability sample survey of ED visits. Each institution participating in NHAMCS was required to supply case records (including temperature) for every *n*-th ED visit after a random start. We analyzed ED visits from the 2003–2010 NHAMCS surveys, which included visits from December 2002–December 2010.^14^,^15^ During this period, 285,798 ED visits were reported to NHAMCS by participating institutions.

Although the Boston dataset mainly includes adults, the national dataset includes many children and infants (demographics: Appendix 1). For better comparability between the datasets, we only included adults (age ≥18, n = 218,574) when analyzing national data in the main paper and most appendices (Appendices 2–5). However, results for younger ages are given in Appendices 6–7.

### Measurements

In the Boston ED, temperatures and measurement times were collected with temporal artery thermometers attached to automatic data-loggers (TAT-5000 model thermometers; Exergen Corporation, Watertown, MA). During the study, 1–4 such thermometers were generally in use. Temperatures were not recorded for all patients, for example, because some simply did not have their temperatures taken. For the national data, the thermometry method was left to the discretion of clinicians and EDs, and is nationally representative of the thermometry methods used at US triages. Temperature measurements were recorded manually on NHAMCS forms.

Common thermometer modalities in EDs include temporal artery, tympanic membrane, oral, rectal, and axillary. There are no strict rules to compare the temperatures taken at these different sites, since each is affected by its own individual benefits and weaknesses of physiology and measurement ease.^18^ Loosely speaking, however, temporal, tympanic, and oral temperatures are often similar, while rectal temperatures are often higher and closer to core temperatures, and axillary temperatures are often the lowest and least similar to core temperatures.^18^,^19^

### Variables

In the Boston study, body temperature was not linked to other hospital records. This was required to preserve patient anonymity, and prevented analysis of potential confounders and age-specific analyses for the Boston data. In the national study, NHAMCS forms included many variables, allowing us to apply multivariable regression to control for 12 potential confounders: gender; age; immediacy to be seen after triage; pain level at triage presentation; race; Hispanic or Latino ancestry; hospital admission; diagnostic or screening services ordered or provided during visit; procedures provided during visit; medications ordered or provided during visit; arrival by ambulance; and expected source of payment (Appendix 2). The purpose of the multivariable regression was to control (account) for changes in the composition of patients who showed up to the ED across the day.

### Definitions

Body temperatures were classified as fever-range vs non-fever range. Various definitions of fever-range temperature are used in practice. To address this, we analyzed several definitions: ≥37.5°C (≥99.5°F), ≥38.0°C (≥100.4°F), ≥39.0°C (≥102.2°F), and ≥40.0°C (≥104.0°F). For the purposes of our study, these categories were termed sub-fever, fever, high fever, and very high fever, respectively. The values were selected from fever thresholds and upper limits of normal appearing in *Rosen’s Emergency Medicine*,^20^ *Tintinalli’s Emergency Medicine*,^21^ and *Harrison’s Principles of Internal Medicine*.^22^

### Data Quality

As a byproduct of automatic data recording, Boston data included accidental measurements taken when thermometers were pointed at floors, walls, and elsewhere, as well as repeated measurements of the same patients. To filter these out we excluded (a) temperatures <35.0°C (<95.0°F) (n = 13,137; 11.4%) because human temperatures are rarely <35.0°C (<95.0°F), and (b) all but the last of any string of temperatures logged by a single thermometer less than 15 seconds apart (n = 15,983; 13.9%). The distribution of intermeasurement times suggested these strings of rapid measurements were likely repeated measurements of the same patient. Further, we excluded records affected by file corruption and other digital collection errors (n *=* 1166; 1.0%). The remaining 93,225 (81.0%) were analyzed.

The national data have the advantage of not including accidental or repeated measurements, since one temperature was recorded per patient manually. However, manual recording led to several disadvantages: First, values clustered at round numbers (e.g., 98.0°F and 102.0°F), suggesting errors in recall and record abstraction (a recognized limitation to NHAMCS^14^,^23^,^24^). Second, measurement times were not recorded. We used patient arrival times as a substitute for our analyses. Third, some visit records lacked temperatures (n = 19,057; 6.7%) or arrival times (n = 3360; 1.2%). These were excluded, leaving 264,617 (92.6%) for analysis. Fourth, thermometer type was not recorded.

Although each data source has limitations, their limitations are different. Despite having different limitations, they both showed the same main findings, supporting the validity of these findings.

### Main Analyses

For both datasets, we analyzed time, body temperature, and body temperature classified as fever range vs non-fever range. Additionally, we analyzed age groups (18–64 and 65+ years) in the national data.

### Sensitivity Analyses

Sensitivity analyses appear in Appendix 3. To confirm that the temperature exclusion criteria were reasonable for the Boston data, we checked that results were unchanged when using other intermeasurement times (5, 30, and 60 sec). We also confirmed that temperatures <35.0°F (<95.0°C) were rare enough in national data (0.3% of temperatures) for their exclusion to be reasonable in the Boston data. Further, to confirm that using arrival times as measurement times was reasonable for the national data, we checked that results were largely unchanged when using times patients were seen instead.

We also investigated the sensitivity of results to differences between weekdays and weekends, autocorrelation in temperature measurements, and differences in the numbers of patients presenting across the day. Principal findings were not changed in any case. Finally, to confirm our results were not attributable to use of temporal artery thermometry, we checked they were similar in national data from 2002–2004, when temporal artery thermometry was rare in EDs.

### Other Analyses

We analyzed national results for pediatric patients (Appendix 6) and infants (Appendix 7). We also evaluated a method proposed by Mackowiak et al to correct the fever threshold for the circadian cycle (Appendix 8).^5^

### Statistical Methods

We analyzed temperature means, temperature standard deviations, and proportions of temperatures in the fever range by time of day. Cases with missing temperature or time were excluded (see *Data Quality* section). For the Boston data, we obtained 95% confidence intervals (CIs) from exact binomial tests or bootstrapping. For the national data, we used different methods because it was necessary to account statistically for the multistage survey design of NHAMCS, as recommended by NHAMCS guidelines.^14^,^15^ Using the R “survey” package,^25^,^26^ we obtained point estimates and 95% CIs from the incomplete beta function for proportions and maximum pseudolikelihood estimation of normal distribution fits for means and standard deviations. For both Boston and national data, smooths of trends in means and standard deviations were obtained using moving averages with approximate inverse-variance weighting.

In national analyses, we also applied multivariable logistic regression to account for the effects on the observed fever incidence of differences in the composition of patients who are triaged at different times of day (Appendix 2). In more technical detail, to allow time-of-day comparisons of the observed incidence of fever while controlling for time-of-day differences in the distributions of 12 patient characteristics, we fit multivariable regressions with the observed incidence of fever as the dependent variable and the 12 patient characteristics as independent variables; we then obtained average marginal predictions by time of day using the approach described by Bieler et al,^27^ as applied in the “survey” package^25^,^26^ using the quasibinomial family. For our age group comparisons, this procedure was modified by removing the controlling for age, but continuing to obtain average marginal predictions over the entire analyzed cohort for other characteristics (i.e., over all ages combined).

## RESULTS

### General Characteristics

Of 115,149 records from the Boston ED, 21,924 (19.0%) were filtered out as described in the methods. We analyzed the remaining 93,225 (81.0%). Of 285,798 records from the nationally representative survey of EDs, 21,181 (7.4%) were excluded due to missing temperature or time values. The remaining 264,617 (93.6%) were analyzed. For the national data, results in the main text and figures are reported for adults only (ages ≥18, n = 202,181), and results for pediatric and infant patients are given in Appendices 6 and 7. Median age was 49 years (interquartile range, 32–66) in the Boston ED and 43 years (interquartile range, 29–59) for adults in the national EDs. Mean body temperature was 36.7°C (98.1°F) in both data sources (95% CI, 36.7–36.7°C, 98.1–98.1°F for both). Patient demographics are summarized in Appendix 1, and temperature distributions are summarized in Appendix 4.

### Fever Incidence Changes Over the Day

**Figure 1:**
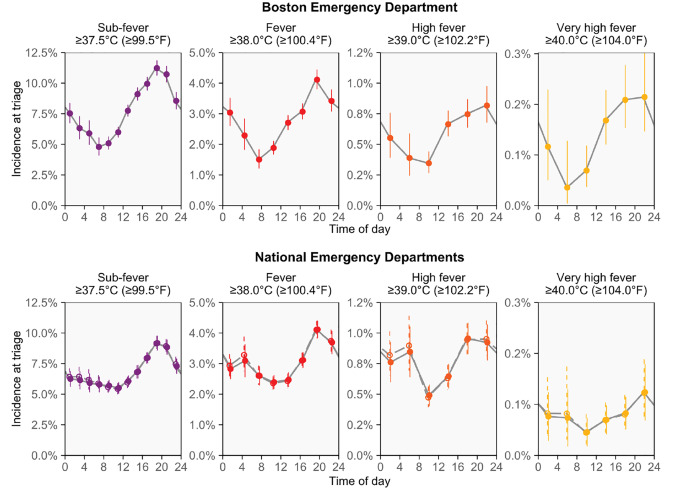
Cyclic changes in the incidence of fever observed across the day. For all investigated definitions of fever, lower fever incidence is observed at morning triages and higher fever incidence is observed at evening triages. The pattern of changing fever incidence resembles the circadian cycle of body temperature and may be caused by it. For the national analyses of US emergency departments, we used multivariable logistic regression to adjust for 12 potential confounders when estimating the incidence of fever observed at triage. Adjusting for the potential confounders led to almost no change in the results; thus, the unadjusted results (hollow points with dashed lines) and adjusted results (solid points with solid lines) often overlap. All confidence intervals are 95%.

Figure 1 shows how the observed incidence of fever changed over the day. In practice, ≥38.0°C (≥100.4°F) may be the most common cut-off used in definitions of fever. Overall, 2.9% of triage temperatures (1 in 35) were in this range at the Boston ED. The percentage of triage temperatures in this range was about 2.5 times higher in the evening as in the morning (7:00–8:59 pm vs 7:00–8:59 am: 4.1% vs 1.6%; risk ratio [RR] 2.5, 95% CI, 2.0–3.3). Similarly large variations were also seen for more extreme definitions of fever: temperatures ≥39.0°C (≥102.2°F) and ≥40.0°C (≥104.0°F) were respectively 2.4- and 3.6-times more common in the evening than in the morning (7:00–8:59 pm vs 7:00–8:59 am: incidences = 0.95% vs. 0.39% and 0.30% vs. 0.08%, respectively; RRs [95% CIs] = 2.4 [1.5–4.3] and 3.6 [1.5–17.7], respectively). For all definitions, the times when fever-range temperatures were least and most common were similar to the times when the circadian cycle results in lowest and highest body temperatures.^1^,^5^

The national data confirmed the presence of strong cyclic variation in the observed incidence of fever, including after using multivariable logistic regression to adjust for 12 potential confounders (Figure 1). For the same fever definitions and times of day mentioned above, the fever incidence observed in the morning and evening were 4.2% and 2.3%, 1.00% and 0.54%, and 0.11% and 0.04%, respectively, while the unadjusted RRs (95% CIs) were 1.8 (1.6–2.1), 1.9 (1.4–2.5), and 2.8 (0.8–9.3), respectively, and the adjusted RRs were 1.8 (1.5–2.1), 1.8 (1.3–2.4), and 2.7 (0.8–9.2), respectively. Overall, the fever incidence changed similarly over the day in the national and Boston data, but the morning decline in fever incidence was not as deep in the national data.

### Relationship Between Fever Incidence and the Daily Cycle of Mean Body Temperature

To help us understand why fever incidence changes so much over the day, we also analyzed diurnal variation in the mean and standard deviation of body temperature. Historically, it is not entirely clear how ≥38.0°C (≥100.4°F) was established as a temperature range for fever. However, the mean plus 2 standard deviations is used as a cut-off to differentiate between normal and abnormal values in many scientific settings, and this value was indeed 38.0°C (100.4°F) in the Boston ED (95% CI, 38.0–38.0°C, 100.3–100.4°F). It was similar in the national data, too (37.9°C, 100.3°F).

**Figure 2:**
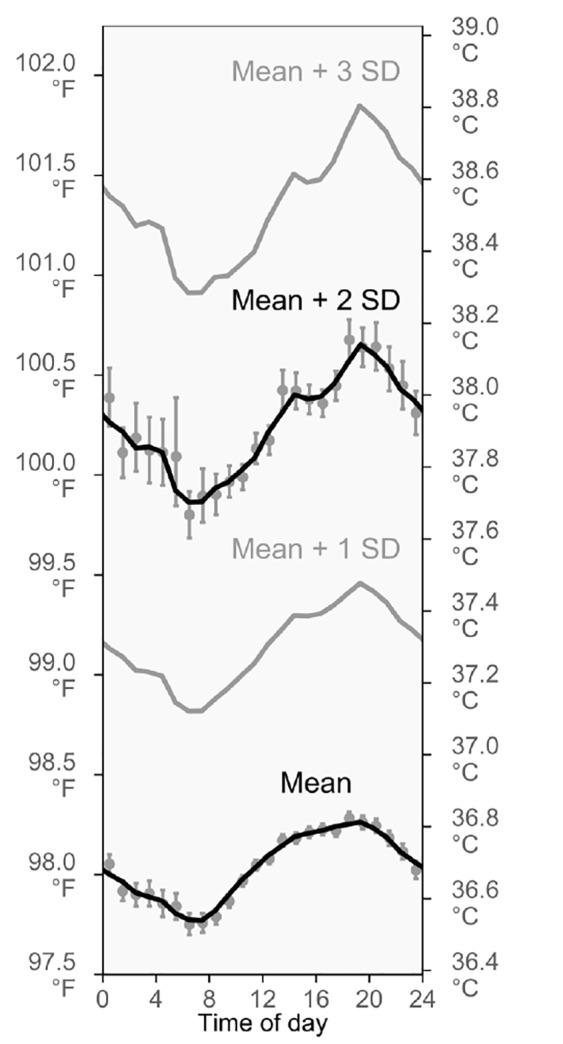
Daily cycles of the mean body temperature at triage, and the mean + 1, 2, and 3 standard deviations. The diurnal pattern of mean body temperature at triage resembles the well-known circadian rhythm of human body temperature. However, we observed that the amplitude of the cycle becomes larger for temperatures that are farther above the mean. Curves are 3-hour moving averages. Error bars are 95% confidence intervals. Results are shown for the Boston emergency department (ED). Similar results for the national EDs are in Appendix 5.

Analysis of both datasets showed that the mean plus 2 standard deviations followed a substantially larger daily cycle than the mean temperature itself (Figure 2, Appendix 5). These findings may help to explain why the time-of-day variations in triage fever incidence (Figure 1) are unexpectedly large: Because daily, cyclic variations are larger for unusually high temperatures than for mean temperatures, the proportion of patients who meet the definition of fever also varies more than would be anticipated based on experience with commonplace temperatures.

### Fever Incidence and Body Temperature in Younger vs Older Adults

To examine how fever incidence was affected by age-associated body temperature changes, we performed comparative analyses of younger adults (ages 18–64, *n* = 163,478) and older adults (ages 65+, *n=* 38,703).

**Figure 3:**
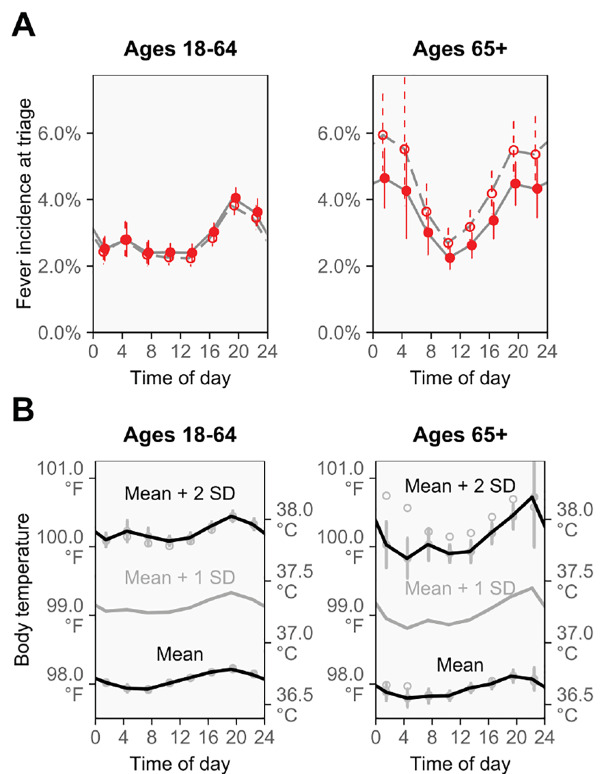
For ages 18–64 and 65+, the cycles of fever incidence and body temperature. (A) The incidence of fever followed large daily cycles in both age groups. Although the older age group had higher fever incidence before adjustment for potential confounders (hollow points and dashed lines), the difference largely disappeared after this adjustment (solid points and solid lines). Fever was defined as body temperature ≥38.0°C (≥100.4°F). (B) Diurnal cycles of body temperature were present in both age groups, with temperatures that were multiple standard deviations above the mean following larger cycles. Mean body temperature was slightly lower in the older age group, both before (hollow points) and after (solid points) adjustment for potential confounders (unadjusted and adjusted difference: 0.1°C [0.1°F]). Results are for national US emergency departments. All confidence intervals are 95%.

As shown in Figure 3A, overall fever incidence was higher in older than younger adults (difference: 1.3 percentage points, 95% CI, 1.0–1.5; ages 18–64, 2.8%; ages 65+, 4.1%), but differences largely disappeared after adjustment for potential confounders (adjusted difference: 0.3 percentage points, 95% CI, 0.0–0.7), which included characteristics related to case severity and use of diagnostic testing (Appendix 2). Fever incidence followed a large daily cycle in both age groups, although older adults appeared to have heightened fever incidence after midnight.

As shown in Figure 3B, mean body temperature was slightly lower in older than younger adults, (difference: 0.1°C [0.1°F], 95% CI 0.1–0.1°C [0.1–0.2°F]; ages 18–64, 36.7°C [98.1°C]; ages 65+, 36.6°C [98.0°C]), a difference that persisted after confounder adjustment (adjusted difference: 0.1°C [0.1°F], 95% CI, 0.0–0.1°C [0.1–0.2°F]). Older adults also had a slightly smaller diurnal cycle of mean body temperature, but temperatures that were multiple standard deviations above the mean followed large diurnal cycles in both age groups.

### Other Analyses

Analyses of pediatric patients showed much higher incidence of fever overall, as well as a different pattern of diurnal temperature variation that reached its minimum at around 8:00–10: am and its maximum after midnight (Appendix 6). Analyses of infants were consistent with an increasing circadian cycle of body temperature during early weeks of life, which matches previous studies^28^,^29^ (Appendix 7).

Appendix 8 investigates a proposal^5^ that recommended correcting for the circadian cycle by changing fever thresholds to >37.2°C (>98.9°F) and >37.7°C (>99.9°F) for oral temperatures taken during mornings and evenings, respectively. In comparison with the common ≥38.0°C (≥100.4°F) fever threshold, the proposal classified more than twice as many patients as having fever (Boston data: 7.4% vs 2.9%; national data: 6.7% vs 3.0%). Additionally, the proposal appeared to overcorrect substantially for the circadian cycle, producing a reversed pattern of much higher incidence of fever during mornings than evenings.

## DISCUSSION

This study establishes that there are large daily cycles in the incidence of fever-range temperatures seen at adult triage. The cycles were observed in a Boston ED and confirmed using a large, nationally representative sample of US EDs. The cycles remained after using multivariable regression to control for 12 potential confounders, and they also continued to be observed in age group comparisons and sensitivity analyses.

In the daily cycles, fever-range temperatures were generally least common at morning triages and most common at triages in the late afternoon and evening. This pattern parallels the usual pattern of diurnal variation of body temperature.^1^,^4^–^6^ Moreover, it is consistent with the longstanding hypothesis that the fixed temperature thresholds for fever are incompatible with the diurnal variation of body temperature, and that the incompatibility causes the detection of fever-causing disease to be artificially diminished in the morning and artificially inflated in the late afternoon and evening.^5^–^9^ Our results provide support for this hypothesis from a real-world healthcare setting. Our results also add to the current understanding by showing that cycles in the observed incidence of fever are larger, and therefore more consequential, than would be anticipated from the diurnal variation of mean body temperature alone.

The large daily cycles in the observed incidence of fever raise the concern that cases of fever-causing disease could be missed or underappreciated in the morning, and that false-positive fevers may be diagnosed in the late afternoon and evening. In practice, then, it is best to evaluate body temperature together with other signs and symptoms of fever, which can include chills and shivering (especially at the start of fever) and sweating (especially at its end).^9^,^30^ The other signs and symptoms of fever may be especially important during mornings, since body temperatures are usually lower at this time and may fail to reach the temperature ranges that are used to identify fever, even when fever is physiologically present. Relatedly, patients who do have fever-range temperatures in the morning may be in worse-than-expected condition because the lower values of morning temperatures in health mean that larger temperature increases are needed to reach the fever range.

Previous studies do not provide enough information to quantify the effects of diurnal body temperature variation on the observed incidence of fever. However, it is possible to take published values and use them in crude, back-of-the-envelope estimates. For example, in analyses of convenience samples, Musher et al^4^ observed that 72% and 84% of patients with fever-causing disease followed an exaggerated version of the usual diurnal cycle of body temperature. So, supposing that three-quarters of patients with fever-causing disease follow an exaggerated version of the usual diurnal cycle, and supposing also that one-third of these patients have temperatures below the ≥38.0°C fever threshold in the morning, then a quarter of all patients with fever-causing disease will not have fever-range temperatures in the morning. On the other hand, using a mean healthy temperature of 36.8°C,^5^ an inter-individual standard deviation of 0.15°C,^31^ and a mean (range) circadian amplitude of 0.25°C (0.05–0.65°C),^5^ simulation suggests that perhaps a quarter of the ostensibly fever-range temperatures recorded at evening triage may be false positives supplied by nonfebrile patients who cross the ≥38.0°C cutoff when reaching highpoints of their healthy circadian rhythms. However, we emphasize that these are crude, back-of-the-envelope estimates, rather than dependable evidence. Their purpose is to illustrate the logic of how the diurnal variations of body temperature are capable of producing large daily cycles in the observed incidence of fever, like those we found. It remains to be determined how much of the cycles are attributable to the lower morning temperatures of patients with fever-causing disease and how much of the cycles are attributable to false-positive fevers in the afternoons and evenings. We leave this to future research.

The incidence of fever seen at triage is not only determined by changes in body temperature, but also by changes in the mix of patients who show up to the ED across the day. In the current study, we applied multivariable regression to account for time-of-day differences in the patient mix seen at triage, which did not remove or reduce the cycle of adult fever incidence, despite including 12 patient characteristics in the analysis. However, it remains possible that the 12 characteristics were not sufficient to control for all important differences in patient mix across the day, and therefore that some of the fever incidence cycle is due to changes in patient mix. (See Appendix 2 for more detail on strengths and limitations of the multivariable approach.) We also observed that the large cycles of fever incidence occurred on both weekdays and weekends (Appendix 3), which suggests the cycles are not a consequence of differences in patient mix associated with people’s work hours or the hours that alternative sources of care are open. Nonetheless, it remains possible that changes in patient mix contribute to the daily cycle of fever incidence and a different study design would be needed to address this possibility conclusively, likely by including many temperatures collected from the same individuals across the day.

Because mean body temperature is lower among older adults (“older is colder”)^13^ and because fever responses can be blunted at older ages,^12^ we also compared findings for 18–64 and 65+ year-olds (Figure 3). Mean body temperature was 0.1°C (0.1°F) lower in the older age group, which is a smaller difference than found in some studies,^12^ but agrees with others, including several large-scale investigations (ages 20–59 – ages 60+: 0.1°C;^13^ 0.02°C reduction per decade;^31^ ages 20–64 – ages 65–95: +0.1°C to −0.1°C, seasonally^32^). Moreover, our results show that the lower mean body temperature of older adults did not translate to lower triage fever incidence, and that the blunted fever responses that have been reported previously^12^ do not eliminate the daily cycle of fever incidence. Instead, fever incidence was higher in the older age group, and only became concordant with incidence at younger ages after statistical adjustment for differences in case mix between the age groups. This suggests that the higher fever incidence among older adults reflected the different ED case mix in this group, instead of being a biological consequence of age.

For all age groups, we suggest investigating how the daily cycle of fever incidence affects diagnosis and outcomes. Triage decisions are upgraded by temperature and other vital signs in an important minority of cases.^33^ We specifically suggest studying sepsis, for which delays in diagnostic maneuvers and management can be especially consequential.^34^ Although body temperature can be an unreliable sign in sepsis,^34^ sub-fever-range body temperature correlates with less-prompt treatment and much greater mortality in sepsis and septic shock.^35^,^36^ For example, among intensive care unit-admitted patients with severe sepsis and septic shock, each 1°C reduction in body temperature was associated with a five percentage-point increase in in-hospital mortality rates, with patients in the highest and lowest temperature brackets having mortality rates of 9% and 50%, respectively.^36^ Seen alongside our results, these findings suggest the hypothesis that lower patient temperatures in mornings could hinder management and perhaps worsen outcomes by delaying recognition of sepsis. It may also be worth accounting for lower morning body temperatures during thermometer-based screenings for outbreaks of fever-causing disease, to reduce the possibility that disease cases are missed during morning screenings.

For both clinical and disease-screening purposes, it may ultimately be worth correcting fever definitions for the diurnal variation of body temperature. To date, one method of correction has been proposed.^5^ The proposal is currently recommended in *Harrison’s Internal Medicine*,^9^ *UpToDate*,^30^ and other medical references, but it appeared to perform poorly in our datasets (Appendix 8). This may be attributable to the small sample size that was originally used to derive the correction, as well as the absence of fevers in the originating study.^5^ Further work should also investigate fever cycles and definitions by age, since we observed differences between adults (main paper), children (Appendix 6), and infants (Appendices 7) in our study, which could also affect corrections. As an alternative to correcting fever thresholds, in some settings it is possible to chart patient body temperature over time and use the appearance of spikes to identify fever, instead of absolute thresholds.

### Generalizability

Because the survey data are nationally representative, the findings likely have good generalizability to US EDs as a whole. However, individual EDs may show somewhat different findings because they use different thermometry methods and serve different populations, which have different age structures, gender ratios, and local climates, each of which can affect body temperature.^37^

## LIMITATIONS

Our study has several limitations. The study design was cross-sectional and there was no patient follow-up. Therefore, we were unable to investigate whether individuals without fever at triage developed it at later times or had it earlier in the day. Similarly, we were unable to evaluate how the temperatures of individuals with fever changed over time. Our analyses do not distinguish between elevated temperatures attributable to fever, hyperthermias (such as heat stroke), or other conditions, and we did not investigate non-temperature symptoms of fever or antipyretics use. For the national data, arrival times had to be used as a surrogate marker for measurement times. Additionally, we did not investigate inter-individual differences in temperature baselines, which depend on age, gender, ovulation, and other characteristics.^31^,^37^ Both of the data sources used in this study also have several limitations (see Methods). However, we note that their limitations are different. Despite having different limitations, they both showed the same main findings, supporting the validity of these findings.

## CONCLUSION

This study of US EDs demonstrates that triage temperatures are lower in the morning than in the afternoon or evening, and that adult patients are much less likely to have triage temperatures that meet the definition of fever in the morning. Clinically, the large difference between the observed incidences of fever during morning and evening triages suggests that it is worth investigating whether the diagnosis, management, and screening of fever-causing diseases are obstructed during mornings, including in cases of sepsis and infectious disease outbreaks.

## Supplementary Information
