# Supplementary material for: Fever Incidence Is Much Lower in the Morning than the Evening: Boston and US National Triage Data
Source: West J Emerg Med. 2020 Jun 24;21(4):909–17. doi: 10.5811/westjem.2020.3.45215 (PMC7390559; doi:10.5811/westjem.2020.3.45215)
Supplement: Supplementary file 1 [file wjem-21-909-s001.pdf]

Online-Only Supplement

for

Fever Incidence is Much Lower in the Morning than the Evening: Boston and US National Triage Data

Charles Harding, Francesco Pompei, Samantha F Bordonaro, Daniel C McGillicuddy, Dmitriy Burmistrov, Leon D Sanchez

Contents **Page**

Appendix 1. Demographics.......... 2

Appendix 2. Controlling for potential confounders.......... 3

Appendix 3. Sensitivity analyses.......... 11

Appendix 4. Figure showing distribution of temperatures by time of day.......... 17

Appendix 5. Figure showing mean and standard deviation in the national data.......... 18

Appendix 6. Results for patients age <18.......... 19

Appendix 7. Results for infants.......... 22

Appendix 8. Investigating Mackowiak et al.'s fever definition.......... 24

References.......... 27

Appendix 1

Supplementary Table 1. General characteristics of emergency department presentations

Characteristic	Boston Emergency Department*		Nationally Representative Survey of Emergency Departments**	
	n (thousands)	Percentage	Unweighted *n* (thousands)	Weighted percentage
**Visits**	110.5	100%	285.8	100%
**Sex**				
**Female**	59.9	54%	153.8	54%
**Male**	50.6	46%	132.0	46%
**Median age (IQR), years**	49	(32–66)	34	(18–52)
**Visits resulting in admission**	40.9	37%	37.9	14%
**Time of visit**				
**10:00 PM – 1:59 AM**	10.4	11%	35.8	13%
**2:00 AM – 5:59 AM**	6.1	5%	16.6	6%
**6:00 AM – 9:59 AM**	10.4	11%	36.2	13%
**10:00 AM – 1:59 PM**	24.3	26%	66.2	23%
**2:00 PM – 5:59 PM**	23.8	26%	64.4	22%
**6:00 PM – 9:59 PM**	19.3	21%	63.2	22%
**Unrecorded**		-	3.4	1%

IQR, interquartile range (25th to 75th percentiles).

* Boston results for visit count, sex, mean age, and rate of admission are for Sept 2009 through Aug 2011. Visit times were assessed from temperature measurement records from Sept 2009 to Mar 2012 (*n* = 93.2 thousand).

** All national results are for emergency department visits that were reported to the survey, from Dec 2002 to Dec 2010. "Unweighted *n*" is the count of survey responses in each category, while "Weighted percentage" is the percentage of emergency department visits in each category, after weighting the results to be nationally representative for the study period. The total *n* for the weighted analysis is 896.3 million, which is an estimate of the total number of emergency departments in the US from Dec 2002 to Dec 2010. When the analyses were limited to individuals age ≥18, the median and IQR age range were 42 and (29–59) years.

APPENDIX 2

Controlling for Potential Confounders

BACKGROUND

We have suggested that the daily cycle of fever incidence is mainly or entirely physiological. However, it is also possible that it is not physiological, and instead reflect changes in the types of patients who present to the emergency department across the day.

To address this, we used multivariable logistic regression and weighting to control for time-of-day changes in the types of patients presenting to the national emergency departments.

METHODS

Analyzed characteristics

Twelve patient characteristics / potential confounders were analyzed:

- Sex: male or female
- Age: years.
- Immediacy to be seen: scaled values from 1-4 and an unknown or no triage category. The questionnaire items for this variable have changed substantially over time in the national survey data. For years 2003-2003, possible values were "less than 15 minutes", "15-60 minutes", ">1 hour to 2 hours", ">2 hours to 24 hours", and unknown or no triage. For 2004-2008, an "immediate" category was added. For 2009-2010, possible values were changed to "immediate", "emergent", "urgent", "semi-urgent", "nonurgent", and unknown or no triage. For our analyses, we coded "less than 15 minutes", "immediate", and "emergent" to 1; "15-60 minutes" and "urgent" to 2; ">1 hour to 2 hours" and "semi-urgent" to 3; and ">2 hours to 24 hours" and "nonurgent" to 4.
- Paint at presentation: none, mild, moderate, severe, or unknown. The questionnaire items for this variable have changed substantially over time in the national survey data. Responses were given as the listed terms until 2008, and an integer value from 0 to 10

afterwards. We recoded responses of 0 as “none”, 1-3 as “mild”, 4-6 as “moderate”, and 7-10 as “severe”, following the survey documentation’s recommendations for 2009.

- Race: black, white, or other.
- Hispanic or Latino ancestry: yes or no
- Admitted to the hospital: yes or no
- Diagnostic or screening services ordered or provided during visit: yes, no, or unknown
- Procedures provided during visit: yes, no, or unknown
- Medications ordered or provided during visit: yes, no, or unknown
- Arrival by ambulance: yes, no, or unknown
- Expected source of payment: Medicaid/SCHIP, Medicare, no charge/charity, private insurance, self-pay, workers Compensation, other, and unknown

We controlled for all 12 confounders in our analyses, with the following exceptions:

1. In the comparisons of adult age groups (18-64 vs. 65+), we did not control for age since the goal was to investigate age-related differences.
2. In the comparisons of infant age groups (first 6 weeks, 6 weeks to 16 weeks, and 16 weeks to 1 year), we did not control for age, for the same reason. Additionally, we did not control for expected source of payment or pain at presentation, for the following reasons:
    - Some of the infant age groups included insufficient data on expected source of payment to analyze, and pain at presentation is often unevaluable in this age group.
    - Additionally, some strata for diagnostic or screening services and procedures were absent from a few of analyzed times of day, and were ignored in the multivariable adjustment.

Statistical approach to confounder control for analyses of the fever incidence recorded at triage

To allow time-of-day comparisons of the observed incidence of fever while controlling for time-of-day differences in the distribution of the patient characteristics, we fit multivariable logistic regressions with the observed incidence of fever as the dependent variable and the patient

characteristics as independent variables. In the regression, each characteristic was treated as a categorical variable except for age, which (when included) was analyzed as a continuous variable using a natural cubic spline (knot locations: ages 25, 35, 45, 55, 65, and 75). No interactions between terms were included in the regressions whose results are shown in this article, but we observed that results did not change substantively when we tried fitting several additional regressions that included interactions between sex, age, immediacy to be seen, race, admitted to hospital, and expected source of payment. The regressions were performed using the quasibinomial family to account for overdispersion. From the multivariable regression fits, we then obtained average marginal predictions of fever incidence by time of day using the approach of Graubard and Korn,[1] as described by Bieler et al.[2] and implemented in the R "survey" package.[3,4]

For our age group comparisons, this procedure was modified by removing the controlling for age, but continuing to obtain average marginal predictions over the entire analyzed cohort for other characteristics (ie, over all ages combined).

Statistical approach to confounder control for analyses of means and standard deviations

For the analyses of time-of-day changes in body temperature means and standard deviations, it was necessary to use a different method of controlling for potential confounders. In particular, the available software packages did not allow us to apply survey methods of multivariable regression to evaluate changes in the temperature standard deviation across the day. Therefore, we used an alternative approach in which we reweighted the national emergency department data to ensure that the distribution of each of the potential confounders was constant across the day and, in particular, was equal to its overall distribution in the dataset as a whole. To be more precise, we raked (iteratively poststratified[3,4]) the national data to reweight it such that that the marginal distribution of each potential confounder was the same during each of eight 3-hour periods of the day, and equaled its overall marginal distribution. The reweighting required that all variables were categorical and, for this reason, we categorized age as 18-29, 30-39, 40-49, 50-59, 60-69, 70-79, 80-89, and 90+.

For a concrete example of the reweighting approach, consider the ages of patients presenting to the emergency department. To control for differences in the ages of patients who presented to the

emergency departments across the day, our dataset was reweighted such that, during each of the analyzed periods of the day, the proportion of patients in each age group was equal to its overall proportion in the dataset as a whole. This reweighting was applied not only for age, but for all of the potential confounders in our analyses, and in such a way that the distributions of all potential confounders were constant across the day in the final reweighted dataset.

After reweighting, temperature means and standard deviations were analyzed by fitting separate Gaussian distributions to temperature records from each hour of the day. Fitting was performed by maximum pseudolikelihood estimation using the R “survey” package.[3]

We also tried applying the reweighting approach to the analyses of fever incidence, in place of the multivariable regression approach. We found that it did not change the results meaningfully.

Methodological issues of observational studies

A well-known issue in observational studies is that the results of analyses can be biased if researchers fail to control for some important confounders. To address this “omitted variables bias” as much as was feasible, we controlled for a wide range of case characteristics in our analyses. Controlling led to little change in our overall results (Figures 1 & 2 of the main article; Appendix 5: Supplementary Figure 5), which suggests to us that controlling for more case characteristics would not modify our findings very much, either. In other words, the wide range of case characteristics that we controlled for, together the similarity between our uncontrolled and controlled findings tends to suggest that the potential for omitted variables bias is relatively small. This is further supported by the similarity between our results for weekdays and weekends (Appendix 3: Supplementary Figures 1 & 2).

Statistical approaches to confounder control are also affected by the general concern that controlling for certain variables can induce bias, rather than remove it. For instance, this could occur if we control for variables that are causal descendants of fever-range temperature. An example of this is “immediacy to be seen.” High body temperatures may lead clinicians to assign a greater immediacy to be seen. Because of this, controlling for immediacy to be seen could inappropriately bias the time-of-day variation in the observed incidence of fever towards the null. On the other hand, time-of-day variations in in immediacy to be seen are also likely to reflect changes in the types of patients who present to emergency departments across the day, which we

wish to control for. So, failure to control for immediacy to be seen could also inappropriately bias the observed incidence of fever (either away from or toward the null). Overall, then, there is no clear answer to the question of whether we should control for immediacy to be seen in our analyses, and the same is true for several other variables that we have considered.

Though the choice of variables that should or should not be controlled for is an important theoretical issue, we note that in the specific case of our study, only very small biases could be induced by controlling for or failing to control for the 12 considered variables. This is because the distribution of each of the variables is actually fairly constant across the day (see Appendix 2: Supplementary Table 2 on the following pages). As a consequence of this, there is little difference between the overall results that we obtained with and without controlling for confounders (Figures 1 & 2 of the main article; Appendix 5: Supplementary Figure 5).

Another statistical concern relevant to our study is selection bias, in the form of Berkson's bias. This concern arises because the study is of individuals presenting to emergency departments, not the general population. At least theoretically, selection bias could induce an artifactual association between time of day and the observed incidence of fever or the observed mean body temperature. This is because both time of day and abnormal body temperature could affect the decision to go to the emergency department: If there are more obstacles to going to the emergency department at some times of day than others, then persons who go at inconvenient times may be more likely to have abnormal temperatures, since a more substantial collection of symptoms would be necessary to get them to make that trip. On the other hand, persons going to the emergency department at convenient times may be relatively less likely to have abnormal body temperatures, since fewer symptoms would be sufficient to get them to make the trip. Altogether, this would induce an inverse association between convenient times of day and the observed incidence of fever, unrelated to the effect of the circadian cycle on fever incidence that we seek to analyze.

Although selection bias is an important theoretical concern, it does not appear to be capable of explaining the large time-of-day variation in adult fever incidence that was recorded at triage in our study, since the observed incidence of fever followed the pattern of being higher at the more convenient times of day (early evening, after work hours) and lower at the less convenient times (after midnight and during work hours). This is the opposite of the pattern that would be induced

by selection bias, and it instead matches the circadian cycle of body temperature. At worst then, the effects of selection bias would be expected to work contrary to the circadian cycle, perhaps biasing results somewhat toward the null—making it appear that the circadian cycle has less of an effect on adult fever incidence than it actually does. Yet, we doubt even this based on the similarity of the results obtained when we did and did not control for the 12 case characteristics, which included characteristics related to severity of symptoms and illness as a whole (for example, "immediacy to be seen" and "admission to hospital"). We also doubt this based on the similarity of results for weekdays and weekends, during which the convenience of going to emergency departments is generally very different.

Whether selection bias could have affected the pediatric results is a more open question that could be worth further study, given the presence of differences between controlled and uncontrolled results in the hours after midnight (Appendix 6: Supplementary Figures 6 & 7).

Results and Discussion

As seen in the main article, there was little difference between the results that we obtained with and without using multivariable regression or reweighting to adjust for differences in the types of adult patients who presented to the emergency departments across the day (Figures 1 & 2 in the main article; Appendix 5: Supplementary Figure 5). This suggests that the large daily cycles in the observed incidence of fever are unlikely to be explained by differences in the types of patients who present to the emergency department across the day.

A cross table was used to directly inspect the distribution of case characteristics across the day (Appendix 2: Supplementary Table 2 on the following pages). This table reveals why using multivariable regression and reweighting to adjust for confounders changed our overall results so little: there were only small differences in the characteristics of the adults who presented to emergency departments at different times of day.

Supplementary Table 2. Distribution of case characteristics across the day, as recorded in the national survey of emergency department visits 2003-2010. Comparing the percentages (black text) across columns shows that the distribution of case characteristics was largely unchanging across the day. Counts data are shown in gray to make it easier to compare the percentages across columns. Results are for individuals aged ≥18. *n* is in thousands.

	Time of day															
	**0:00-2:59**		**3:00-5:59**		**6:00-8:59**		**9:00-11:59**		**12:00-14:59**		**15:00-17:59**		**18:00-20:59**		**21:00-23:59**	
**Variable**	Unwtd. *n*	Wtd. %	Unwtd. *n*	Wtd. %	Unwtd. *n*	Wtd. %	Unwtd. *n*	Wtd. %	Unwtd. *n*	Wtd. %	Unwtd. *n*	Wtd. %	Unwtd. *n*	Wtd. %	Unwtd. *n*	Wtd. %
Female	7.1	53.9%	4.6	52.2%	9.2	54.3%	20.3	57.0%	20.8	57.9%	19.6	57.5%	18.7	58.2%	13.6	57.7%
Age group, yrs																
18-29	4.5	34.2%	2.6	29.1%	3.8	22.2%	7.9	22.0%	8.9	24.9%	9.0	26.4%	9.4	29.5%	7.8	32.9%
30-39	2.6	19.0%	1.6	17.7%	3.1	18.1%	6.2	17.1%	6.3	17.2%	6.1	17.8%	6.0	18.7%	4.6	19.0%
40-49	2.2	16.8%	1.6	17.4%	3.3	18.8%	6.6	17.9%	6.6	18.1%	6.2	17.9%	5.6	16.9%	4.1	16.8%
50-59	1.6	11.5%	1.2	13.1%	2.6	15.0%	5.1	14.2%	5.0	13.6%	4.7	13.7%	4.3	13.3%	2.9	12%
60-69	0.9	7.1%	0.8	8.7%	1.6	9.3%	3.5	9.8%	3.3	9.0%	3.0	8.9%	2.6	8.2%	1.8	7.7%
70-79	0.8	6.1%	0.6	7.5%	1.4	8.4%	3.2	9.4%	3.1	8.6%	2.5	7.4%	2.1	6.6%	1.4	6.0%
80-89	0.6	4.2%	0.4	4.9%	1.1	6.6%	2.7	7.5%	2.5	6.8%	2.1	6.2%	1.7	5.4%	1.0	4.2%
90+	0.1	1.0%	0.1	1.5%	0.3	1.5%	0.7	2.1%	0.7	1.8%	0.6	1.8%	0.4	1.4%	0.3	1.4%
Race																
Black	3.3	24.3%	2.3	24.7%	4.2	24.6%	8.3	22.8%	8.0	21.5%	7.4	21.2%	7.0	21.1%	5.5	22.6%
White	9.4	72.3%	6.2	71.4%	12.2	72.2%	26.2	73.9%	26.8	75.3%	25.4	75.4%	23.9	75.6%	17.3	73.8%
Other	0.6	3.4%	0.5	3.9%	0.7	3.2%	1.6	3.2%	1.6	3.2%	1.5	3.4%	1.4	3.4%	1.1	3.5%
Hispanic or Latino																
Yes	1.8	11.8%	1.2	12.8%	2.1	11.2%	4.4	11.0%	4.5	11.1%	4.2	11.1%	4.1	11.5%	3.2	12.2%
No	11.5	88.2%	7.7	87.2%	15.0	88.8%	31.6	89.0%	31.8	88.9%	30.1	88.9%	28.2	88.5%	20.8	87.8%
Immediacy to be seen																
1: immediate+emerg	2.3	16.4%	1.6	17.5%	2.7	15.2%	5.5	15.0%	5.5	14.8%	5.0	14.5%	4.8	14.4%	3.8	15.2%
2: urgent	5.3	40.6%	3.6	41.2%	6.8	40.7%	14.1	39.7%	14.4	40.1%	13.5	40.4%	12.8	40.1%	9.5	40.4%
3: semi-urgent	2.8	23.2%	1.8	21.4%	3.8	22.5%	8.4	24.2%	8.4	23.9%	8.1	24.2%	7.4	24.1%	5.4	23.3%
4: non-urgent	1.1	8.7%	0.8	7.9%	1.7	10.1%	3.7	10.1%	3.7	10.1%	3.4	9.6%	3.1	9.8%	2.2	9.3%
Unknown	1.6	11.2%	1.1	12.0%	2.2	11.5%	4.3	11.0%	4.4	11.1%	4.3	11.3%	4.1	11.6%	3.1	11.8%
Pain at presentation																
None	2.4	17.2%	1.7	18.1%	3.1	17.2%	7.1	18.8%	7.2	18.6%	6.5	17.6%	5.7	16.8%	4.3	17.0%
Mild	1.5	11.4%	1.0	10.4%	2.2	12.1%	4.7	12.4%	4.7	12.3%	4.3	12.2%	4.0	11.7%	2.9	11.4%
Moderate	2.7	20.8%	1.9	21.5%	3.9	23.2%	8.4	23.6%	8.3	22.9%	7.9	23.3%	7.6	23.3%	5.5	22.7%
Severe	3.7	29.8%	2.5	30.3%	4.5	28.0%	8.7	26.0%	8.9	26.4%	8.5	27.1%	8.4	28.2%	6.3	28.0%
Unknown	2.9	20.9%	1.8	19.7%	3.4	19.5%	7.2	19.3%	7.3	19.8%	7.0	19.8%	6.6	20.0%	5.1	21.0%
Arrival by ambulance																
Yes	3.1	22.3%	2.2	25.0%	3.1	17.4%	5.9	16.7%	6.3	17.2%	5.9	17.0%	5.5	16.8%	4.5	18.1%
No	9.6	73.7%	6.3	70.9%	13.4	78.7%	28.5	79.0%	28.3	78.4%	26.7	78.3%	25.4	78.8%	18.4	77.4%
Unknown	0.6	3.9%	0.4	4.1%	0.7	3.8%	1.6	4.3%	1.7	4.4%	1.7	4.7%	1.4	4.4%	1.1	4.5%

Supplementary Table 2 (continued).

Variable	Time of day															
	**0:00-2:59**		**3:00-5:59**		**6:00-8:59**		**9:00-11:59**		**12:00-14:59**		**15:00-17:59**		**18:00-20:59**		**21:00-23:59**	
	Unwtd. *n*	Wtd. %	Unwtd. *n*	Wtd. %	Unwtd. *n*	Wtd. %	Unwtd. *n*	Wtd. %	Unwtd. *n*	Wtd. %	Unwtd. *n*	Wtd. %	Unwtd. *n*	Wtd. %	Unwtd. *n*	Wtd. %
Any test or diagnostic																
Yes	10.5	78.5%	7.3	80.4%	13.8	79.7%	28.7	79.8%	29.0	79.2%	27.1	79.1%	25.4	78.2%	18.9	78.3%
No	2.5	20.5%	1.6	18.5%	3.2	19.4%	6.9	19.2%	7.0	19.8%	6.8	20%	6.5	20.8%	4.8	20.4%
Unknown	0.1	1.0%	0.1	1.1%	0.2	0.9%	0.4	1.0%	0.4	1.0%	0.3	0.9%	0.3	1.1%	0.3	1.3%
Any procedure																
Yes	6.8	51.0%	4.7	53.5%	8.6	50.7%	17.9	49.9%	17.8	49.4%	17.1	50.1%	16.1	50.1%	11.8	49.4%
No	5.9	45.0%	3.9	42.6%	7.9	45.3%	16.7	45.9%	17.1	46.5%	15.8	45.7%	14.8	45.9%	11.1	46.2%
Unknown	0.5	4.0%	0.3	3.9%	0.7	4.0%	1.4	4.2%	1.5	4.1%	1.4	4.2%	1.3	4.0%	1.0	4.4%
Any medication																
Yes	10.8	81.3%	7.3	82.5%	14.0	82.8%	28.7	80.2%	28.6	79.3%	27.2	80.1%	26.1	81.3%	19.4	81.2%
No	2.4	17.8%	1.5	16.6%	3.0	16.3%	7.0	18.9%	7.3	19.4%	6.7	18.7%	5.9	17.6%	4.3	17.7%
Unknown	0.1	0.9%	0.1	0.9%	0.1	0.8%	0.3	0.9%	0.4	1.3%	0.4	1.2%	0.3	1.0%	0.2	1.1%
Admitted to hospital																
Yes	2.0	14.7%	1.6	16.9%	2.6	15.5%	5.7	15.9%	5.9	16.1%	5.6	16.1%	4.8	14.6%	3.5	14.4%
No	11.2	85.3%	7.4	83.1%	14.5	84.5%	30.3	84.1%	30.4	83.9%	28.7	83.9%	27.5	85.4%	20.5	85.6%
Expected form of payment																
Medicaid/SCHIP	2.7	19%	1.7	17.4%	3.0	15.8%	6.8	17.0%	7.3	18.0%	6.9	18.7%	6.6	18.8%	5.0	19.7%
Medicare	2.2	16.4%	1.6	19.0%	3.6	21.7%	8.3	23.8%	8.1	22.6%	7.0	20.9%	6.0	18.9%	4.0	16.8%
No charge/charity	0.1	1.1%	0.1	1.2%	0.3	1.7%	0.4	1.2%	0.4	1.3%	0.3	1.1%	0.3	0.9%	0.3	1.0%
Private insurance	4.3	33.3%	3.0	34.0%	5.9	34.8%	11.5	32.4%	11.0	31.2%	10.9	32.3%	10.8	33.9%	8.0	34.0%
Self-pay	2.5	19.9%	1.6	18.5%	2.7	16.3%	5.5	16.2%	5.8	16.9%	5.6	17.0%	5.5	17.8%	4.3	18.7%
Workers Compensation	0.2	1.5%	0.1	1.3%	0.4	2.2%	0.8	2.0%	0.7	1.9%	0.7	2.2%	0.6	1.7%	0.3	1.4%
Other	0.4	2.7%	0.2	2.7%	0.4	2.3%	0.8	2.2%	1.0	2.7%	0.9	2.7%	0.8	2.5%	0.6	2.5%
Unknown	0.9	6.0%	0.5	5.8%	0.9	5.2%	1.9	5.2%	2.0	5.5%	1.9	5.2%	1.8	5.4%	1.4	5.8%

Unwtd. n gives the unweighted count of survey responses in thousands. *Wtd. %* gives the percentage weighted to be nationally representative of emergency department visits in the US by adults (ages ≥18 years).

APPENDIX 3

Sensitivity Analyses

Several analyses were performed to investigate the sensitivity of our main findings to the assumptions used in our main analyses, such as the inclusion/exclusion criteria and the statistical assumptions. None of the sensitivity analyses showed importantly different findings than the main analyses.

- In our main analysis of the Boston data, we excluded all but the last of any chain of temperatures taken less than 15 seconds apart, as a means of removing repeated temperatures taken from the same patients. To check the sensitivity of our results to this filtering process, we repeated all analyses of the Boston data using 5, 30, and 60 seconds instead. None of our results were meaningfully changed in these sensitivity analyses.
- Although exact measurement times were available for the Boston data, the national survey only included patient arrival times and the times that patients waited to be seen. For the main analyses, we used the patient arrival times because recorded waiting times were missing or appeared imprecise in some cases. As a sensitivity analysis, however, we repeated our analyses of the national data using the sum of the patient arrival time and waiting time. No substantial differences were seen from the main analysis.
- None of our statistical analyses accounted for autocorrelation of temperature measurements, which was present in most regression analyses to an extent that was small, but statistically significant given the large sample size of our study. For our Boston analyses of the daily cycle in mean body temperature and the standard deviation of body temperature, it was observed that including a continuous autoregressive process of order 1 did not remove or substantially reduce residual autocorrelation. Other options for accounting for autocorrelation were made impractical by the large data sizes.

  Therefore, instead of accounting for autocorrelation, we performed sensitivity analyses to investigate how autocorrelation could have affected our Boston results. Specifically, we repeated the analyses after sampling subsets of temperatures under the condition that no two temperatures taken in the same emergency department were within 10 minutes (first sensitivity analysis), 1 hour (second sensitivity analysis), or 3 hours (third

sensitivity analysis) of each other. The 10-minute sensitivity analysis did not result in meaningful changes to the study findings. The 1- and 3-hour sensitivity analyses did not result in meaningful changes in study findings except for very high temperatures (roughly ≥102.2°F, ≥39.0°C), for which sample sizes were so reduced by the sensitivity analyses that results became highly uncertain and uninterpretable due to broad confidence intervals.

Despite excluding temperatures taken at nearby times, mild residual autocorrelation remained in the sensitivity analyses. Further investigation showed that autocorrelation was reduced, although not eliminated, when the number of knots in spline fits was increased. We attribute the persistent, minor autocorrelation to the very large samples sizes and mild underfitting of our regression models, which was preferred to no underfitting because it serves as a protection against overfitting the noisy temperature datasets.

- Patients are much more likely to present to emergency departments at some times of day than at others. To investigate the sensitivity of our findings to this time-of-day variation, we down-sampled the datasets such that the rate of presentation was similar at all times of day. Particularly, we calculated the rate of temperature measurement for the hour of the day during which the fewest temperatures were taken (Boston data: 1125 temperatures at 4-5 AM; national data: 3464 at 4-5 AM), and took random samples of data collected at all other hours such that the frequency of temperature measurements was constant across the day. Our analyses were then repeated. This sensitivity analysis substantially reduced the total sample sizes in our study, and therefore left the findings uncertain for very high temperatures, as previously. Otherwise, however, it did not result in meaningful changes to the study findings.
- As a check of the influence of work hours on our findings, we also compared weekend vs. weekday results for the Boston and national data.

  Work hours may affect the decision of febrile individuals to go to the emergency department because they may prefer not to leave work, and because alternatives to the emergency department (clinics, primary care offices, and etc.) are more likely to be open during the day. Both of these factors could artificially deflate the observed incidence of fever at emergency department triage during the day. However, on weekends, individuals

are less likely to be working and alternatives to the emergency department are less likely to be open. We therefore reasoned that, if work hours had large effects on fever incidence at triage, then there should be large differences between weekdays and weekend results, with the cycles in the observed incidence of fever being substantively reduced during weekends.

The observed incidences of fever during weekdays and weekends are shown on the following pages (Appendix 3: Supplementary Figures 1 & 2). Inspection showed only minor differences between weekdays and weekends, supporting our conclusion that the observed daily cycle of fever incidence is likely to be physiological phenomenon, rather than a reflection of changes in who decides to go to emergency departments.

As in several of the previous sensitivity analyses, however, reduced sample sizes led to uncertain results for high fevers and very high fevers.

- We also performed analyses to check that our study findings were not due to the use of temporal artery thermometry. In the Boston data, temperatures were collected using temporal artery thermometers only. In the national data, the method of temperature collection (oral, temporal, tympanic, rectal, or etc.) was left to the discretion of clinicians and emergency departments, and was not recorded on survey forms.

  Because temporal artery thermometers were rare during the early years of the national survey, we decided to repeat our analyses on these years only to check that our findings were not attributable to temporal artery thermometry. We analyzed the year 2003 and 2004 NHAMCS surveys, which include emergency department records from December 2002 to December 2004. Results are shown on the following pages (Appendix 3: Supplementary Figure 3). As in the previously mentioned sensitivity analyses, we found no meaningful changes to the study findings, but the reduced sample sizes made our results uncertain for very high fevers.

In summary, multiple sensitivity analyses were conducted to check potential sources of bias. The results of the sensitivity analyses were consistent with our main findings.

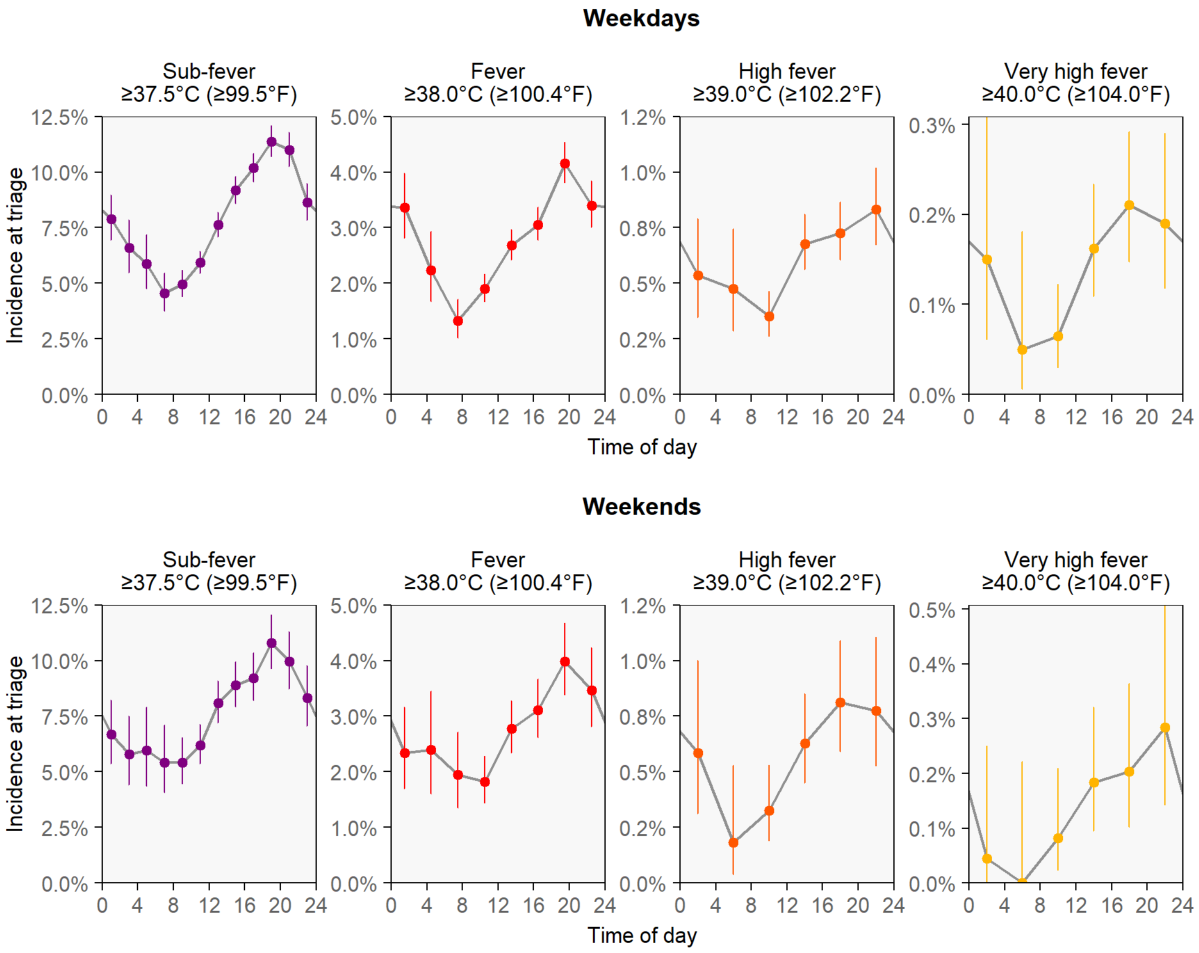

Supplementary Figure 1. Boston emergency department: observed incidence of fever by time of day at triage, as shown for weekdays and weekends separately.

Large daily cycles in the observed incidence of fever occurred during both weekdays and weekends. Although some differences were seen between the weekday and weekend results, the differences were generally small in comparison with the confidence intervals. Confidence intervals are 95%.

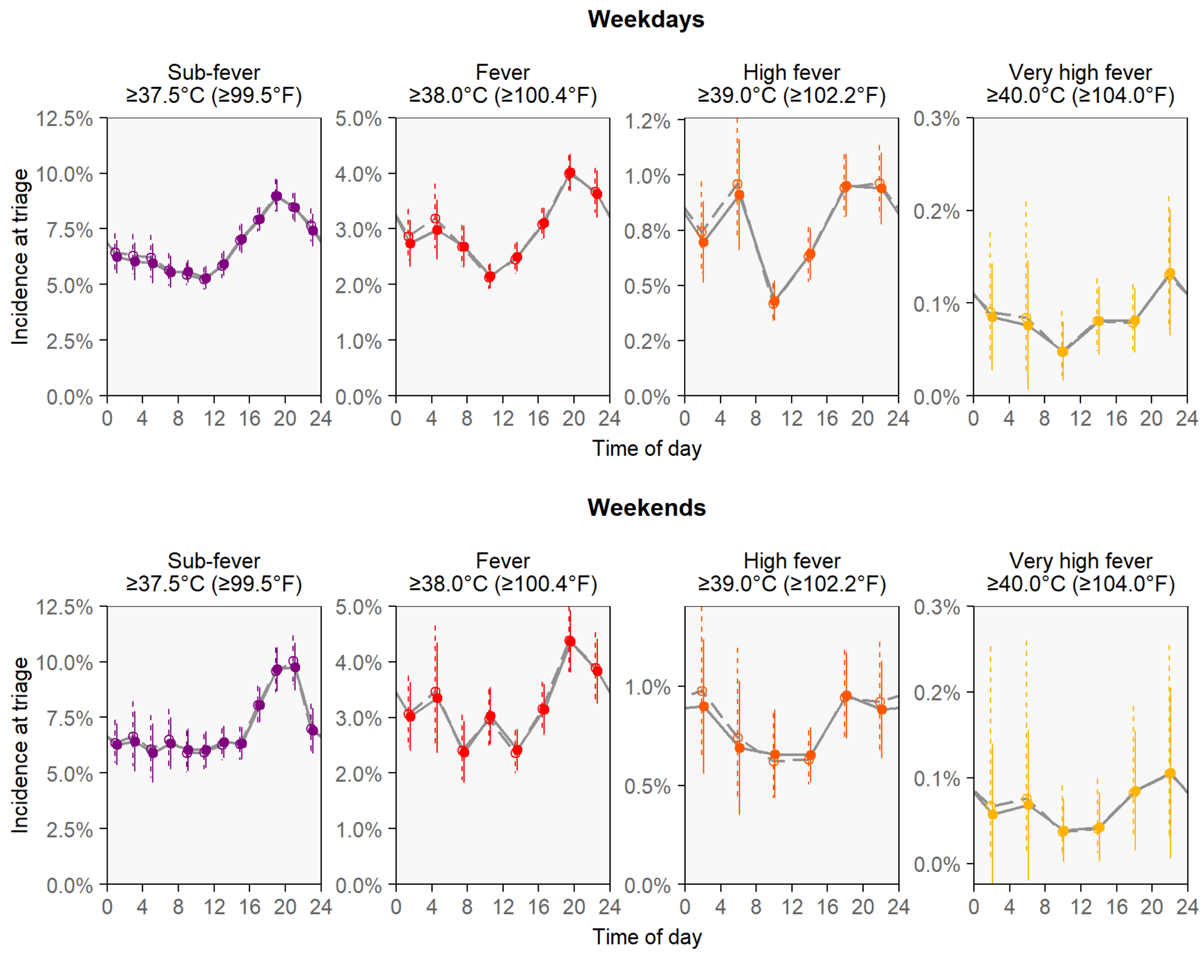

Supplementary Figure 2. National emergency departments: observed incidence of fever by time of day at triage, as shown for weekdays and weekends separately.

Large daily cycles in the observed incidence of fever occurred during both weekdays and weekends. Although some differences were seen between the weekday and weekend results, the differences were generally small in comparison with the confidence intervals. Results obtained before controlling for potential confounders are shown by hollow points and dashed lines, while results obtained after controlling are shown by solid points and solid lines. Confidence intervals are 95%. NHAMCS documentation warns that estimates from the survey should be considered unreliable if the standard error is more than 30% of the estimated value. Based on this criterion, we caution that the reported incidence of very high fevers at triage should not be relied upon for the weekends or the mornings of weekdays.

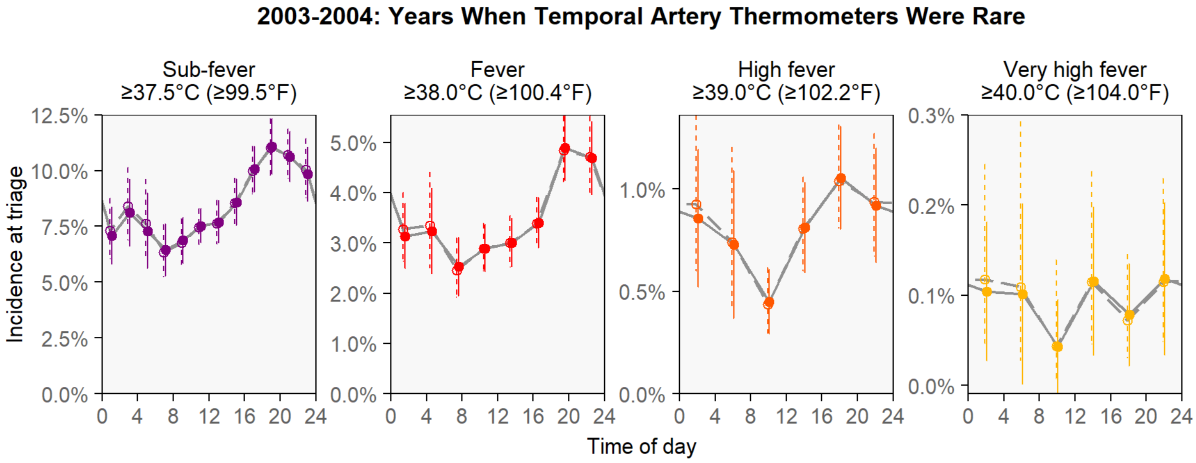

Supplementary Figure 3. National emergency departments: observed incidence of fever by time of day at triage, as shown for the period of the study when temporal artery thermometers were rare in the emergency department setting.

We decided to repeat our analyses on data collected from the national emergency departments in the earliest years of our study (December 2002 to December 2004), when use of temporal artery thermometers was rare in emergency departments. The results were similar to our main analyses, confirming that the observed daily cycle of fever incidence is not attributable to the use of temporal artery thermometers. However, the reduced sample sizes made our results uncertain and potentially unreliable for very high fevers: according to NHAMCS documentation, estimates from the survey should be considered unreliable if the standard error is more than 30% of the estimated value. Results obtained before controlling for potential confounders are shown by hollow points and dashed lines, while results obtained after controlling are shown by solid points and solid lines. All confidence intervals are 95%.

APPENDIX 4

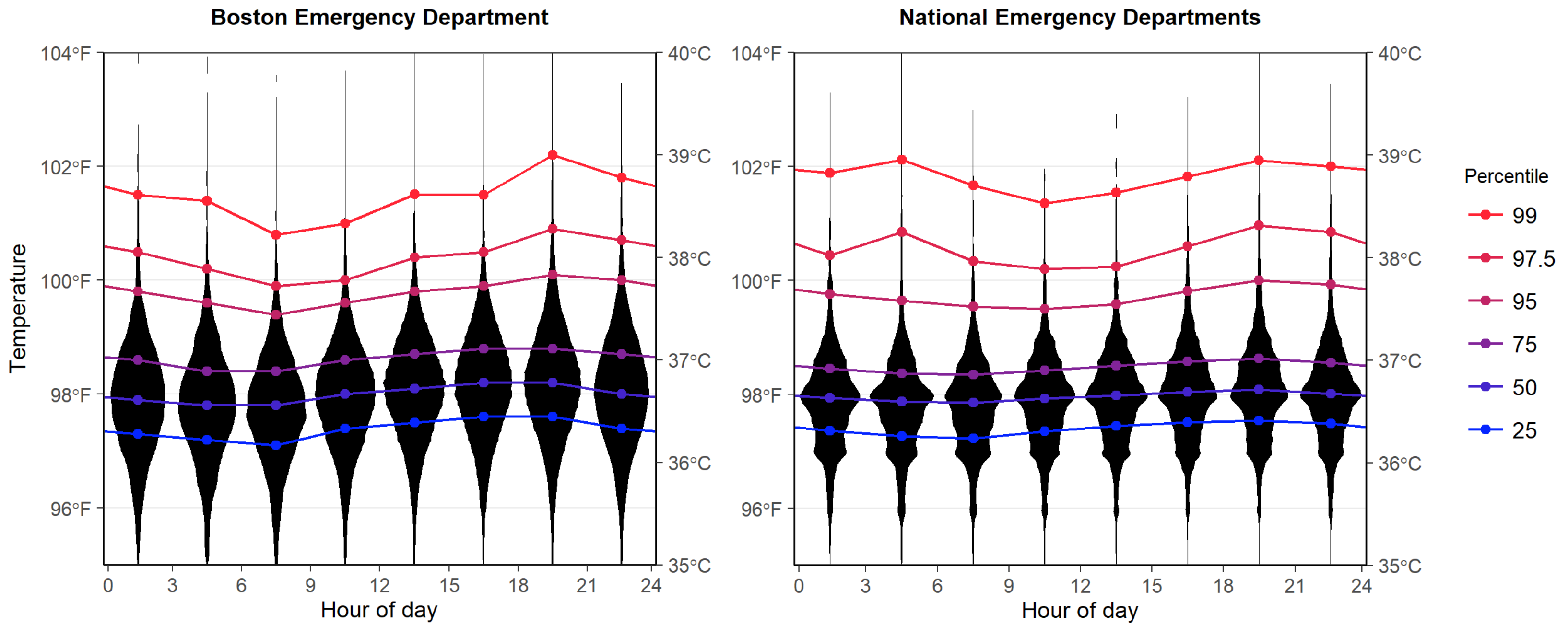

Supplementary Figure 4. Violin plots showing the distribution of temperatures measured across the day. For each 3-hour period of the day, the black region (the "violin") shows the distribution of temperature measurements: the width of this region is proportional to the number of measurements at the indicated temperature. As can be seen, the temperatures from the Boston emergency are smoothly distributed at all times of day. However, the national emergency department data show clustering of recorded temperatures at some values, suggesting the presence of some chart recording or abstraction errors. Superposed over the violins are percentiles of the temperature distribution. The difference in scale between the daily cycles of high and typical temperatures is evident from the different diurnal patterns in the high (95, 97.5, 99) versus typical (25, 50, and 75) percentiles of the temperature distribution. At the scale of y-axis, the daily cycle is clearly visible for high percentiles, but is barely evident for typical percentiles. The Boston emergency department data are from 2009-2012. The national emergency department data are from 2002-2010 and are limited to adults (ages ≥18). They are nationally representative of US emergency departments for this age range. For the Boston data, results were obtained by computing quantiles directly. For the national data, results were obtained using quantile estimation for sample surveys with the R "survey" package.

Appendix 5

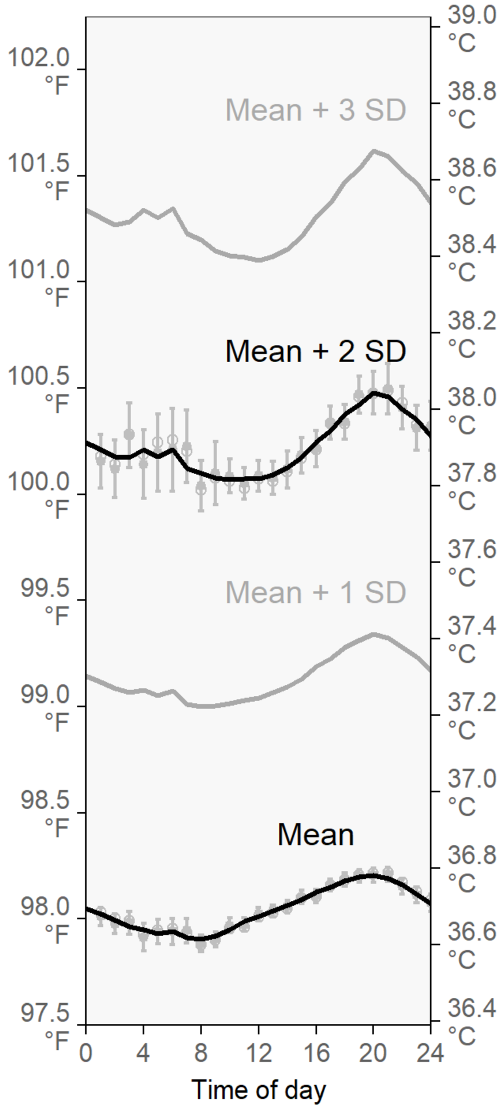

Supplementary Figure 5. National survey results: daily cycles of mean body temperature for adult patients at triage, and the mean + 1, 2, and 3 standard deviations (SDs). The times of minimum and maximum body temperatures are similar in the national and Boston emergency departments. Additionally, in both data sources, the amplitude of the daily cycles become larger for temperatures that are further above the mean. However, the cycles are somewhat smaller in the national data than in the Boston data, and the national data do not show the early afternoon peak that is evident in the Boston data.

Results obtained with and without controlling for potential confounders are shown by the solid and hollow points, respectively. There was almost no difference between the two analyses. 95% confidence intervals are shown for the results obtained while controlling for potential confounders. The confidence intervals and points account for the NHAMCS survey design, and were obtained with the R survey package by fitting separate Gaussian distributions to temperature records from each hour of the day. Curves are 3-hour moving averages of the results obtained while controlling for potential confounders.

The results shown in the figure are limited to patients aged ≥18. Results for pediatric and infant patients can be found in Appendix 6: Supplementary Figure 7 and Appendix 7: Supplementary Figure 8B.

APPENDIX 6

Results for Patients Age 1-17

In the main article, results for the national emergency departments are limited to ages ≥18 to make the age distribution of included cases more similar to those encountered in the Boston emergency department, and thereby allow the national emergency department results to serve as a check of the Boston data.

This appendix reports the results for the national emergency departments at ages 1-17 (n=54,004), for completeness. Mean body temperature was 37.1°C (98.7°F) with a 95% CI of 37.1-37.1°C (98.7-98.9°F), which was substantially higher than the mean value of 36.7°C (98.1°F) observed at ages ≥18. The higher mean body temperature appeared to be largely due to the very high incidence of fever in the pediatric patients, which is as expected because fever is one of the most common complaints for pediatric presentations at emergency departments.[5]

It was also observed that the daily cycles of fever and body temperature followed different patterns in adult and pediatric patients who present to the emergency department: although the minimum incidence of fever at triage was recorded during the morning for both adult and pediatric patients, the maximum incidence of fever at triage was generally recorded in the evening for adult patients and after midnight or during the very early morning for pediatric patients.

In future studies, it may be valuable to investigate additional age groups.

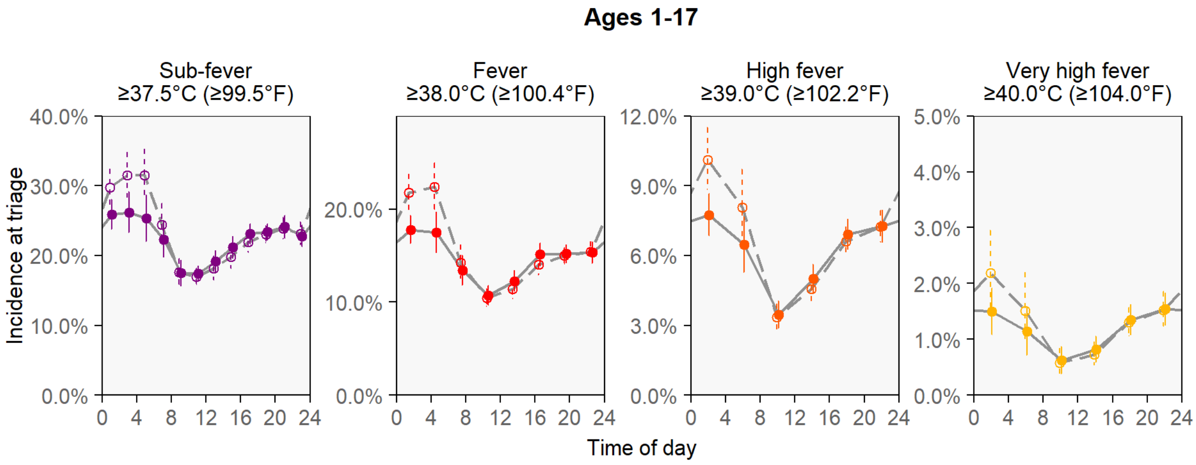

Supplementary Figure 6. This figure presents the same analysis as Figure 1 of the main text, but for ages 1–17 instead of ≥18. For triaged patients ages 1–17, fever is much more common overall. The minimum fever incidence was observed at about 8:00-10:00 AM, while the maximum fever incidence was observed after midnight. Results obtained before controlling for potential confounders are shown by hollow points and dashed lines, while results obtained after controlling are shown by solid points and solid lines. Confidence intervals are 95%.

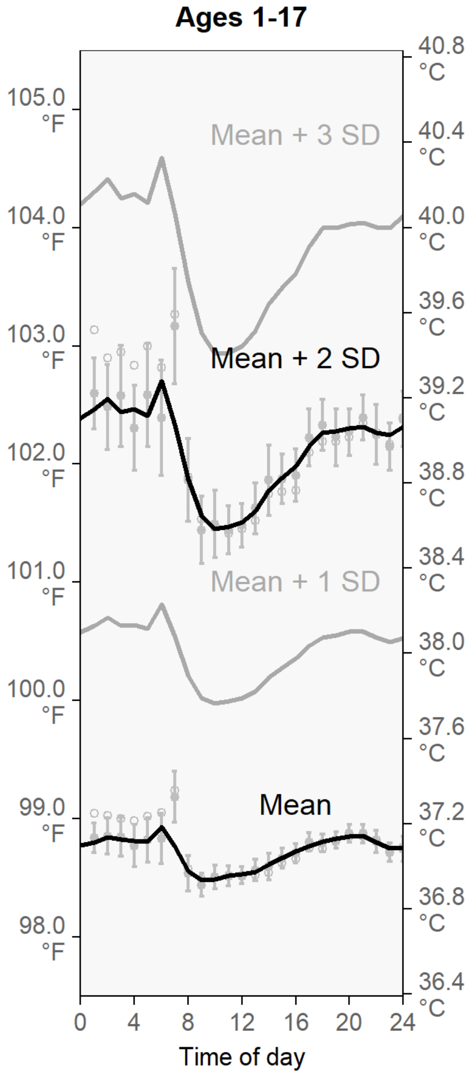

Supplementary Figure 7. National survey results: daily cycles of body temperature at triage for patients age 1–17. The figure shows the mean body temperature and the mean + 1, 2, and 3 standard deviations (SDs). A similar analysis of patients aged ≥18 can be found in Appendix 5: Supplementary Figure 5.

The daily cycle of body temperature and the standard deviation were much larger at ages 1–17 than at ages ≥18, potentially as a consequence of the higher triage fever incidence in pediatric emergency department patients. Further, the diurnal pattern of temperature variation was different at the ages 1–17, reaching its minimum at around 8:00-10:00 AM and its maximum after midnight and during the early or middle morning.

Results obtained with and without controlling for potential confounders are shown by the solid and hollow points, respectively. 95% confidence intervals are shown for the results obtained while controlling for potential confounders. The confidence intervals and points account for the NHAMCS survey design, and were obtained with the R survey package by fitting separate Gaussian distributions to temperature records from each hour of the day. Curves are 3-hour moving averages of the results obtained while controlling for potential confounders.

APPENDIX 7

Results for Infants

In infants, the circadian cycle of body temperature is minimal during the first 6 weeks and grows rapidly thereafter, becoming fully established by about 16 weeks of age.[6–8] We analyzed fever incidence and body temperatures at infant ages (<1 year old; n=8426) to check whether the establishment of the circadian cycle was evident in the emergency department data. The following age groups were analyzed: first 6 weeks (n=1146), 6 weeks – 16 weeks (n=1515), and 16 weeks – 1 year (n=5765).

Before 6 weeks of age, it was difficult to determine whether fever incidence cycled because of because of prohibitively wide confidence intervals, but the daily cycle of fever incidence was fully established by the 16 weeks – 1 year age group (Appendix 7: Supplementary Figure 8A). Overall fever incidence was very high in infants, likely because fevers are a main reason for emergency care for infants.[5] The comparatively lower fever incidence in the first 6 weeks may be explained by blunted fever responses in newborns.[9]

There was no clear diurnal cycle of mean body temperature during the first 6 weeks of age, consistent with previous research,[6–8] although results were again difficult to interpret due to wide confidence intervals (Appendix 7: Supplementary Figure 8B). By the 16 weeks – 1 year age group, the daily cycle of body temperature was clearly established, which is also consistent with previous findings.[6–8] Mean body temperature was high in the 16 weeks – 1 year age group, likely also reflecting a high prevalence of fevers.

Overall, analyzing infant triage temperatures showed results consistent with the establishment of the circadian cycle of body temperature, though interpretation was sometimes difficult due to wide confidence intervals.

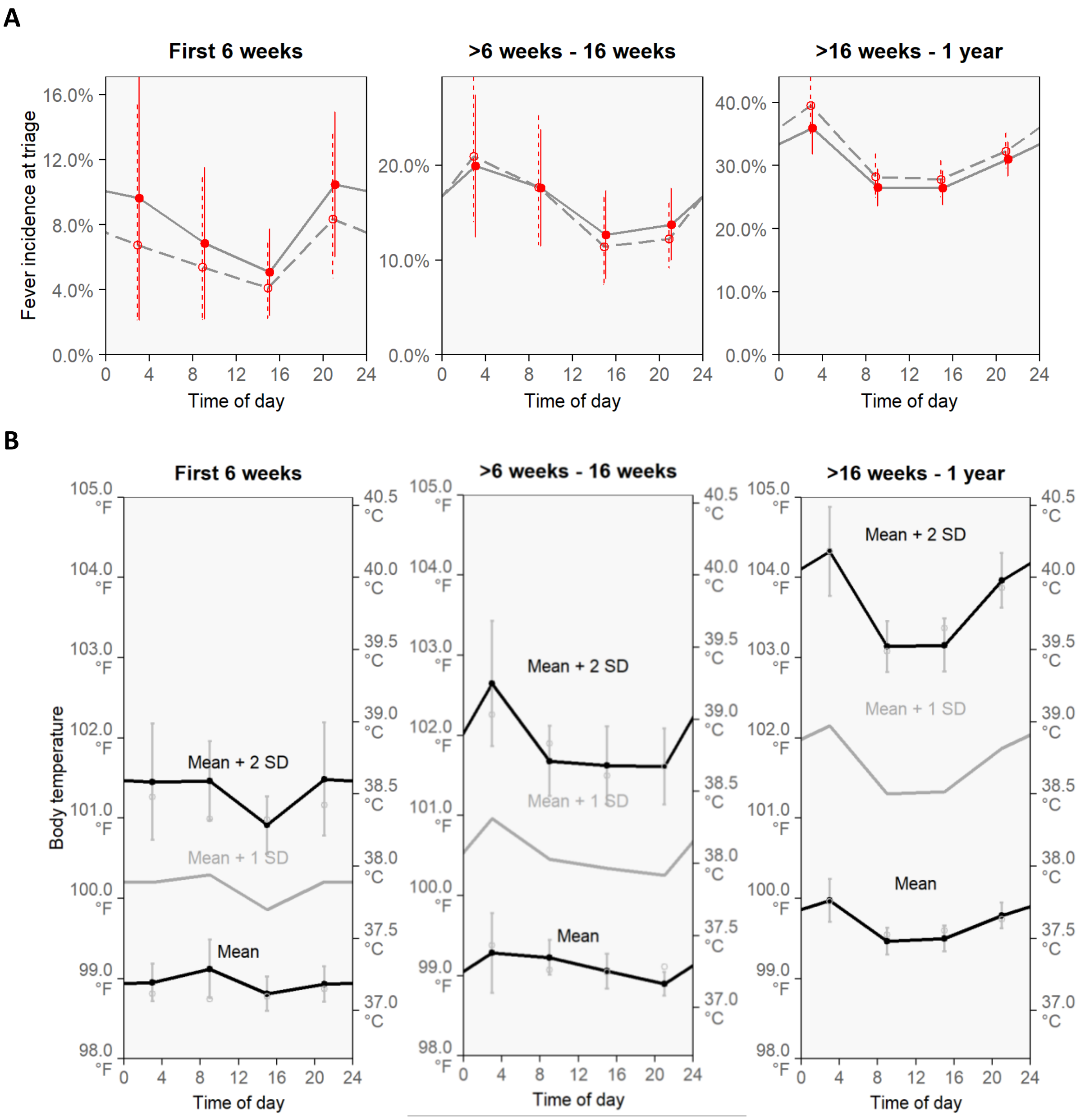

Supplementary Figure 8. For infants, analyses of the time-of-day patterns of fever incidence and body temperature. (A) Before 6 weeks of age, it was difficult to determine whether fever incidence cycled because of because of prohibitively wide confidence intervals, but the daily cycle of fever incidence was fully established by 16 weeks – 1 year. Fever was defined as body temperature ≥38.0°C (≥100.4°F). (B) During the first 6 weeks of age, there was no clear diurnal cycle of mean body temperature, consistent with previous studies of newborn and infant development. By the 16 weeks – 1 year age group, the daily cycle of body temperature had become established, which is also consistent with previous findings. Mean body temperature was high in the 16 weeks – 1 year age group, likely reflecting high prevalence of fevers. Results are for national US emergency departments. Results obtained before controlling for potential confounders are shown by hollow points and dashed lines, while results obtained after controlling are shown by solid points and solid lines. All confidence intervals are 95%.

APPENDIX 8

Investigating Mackowiak et al.'s Fever Definition

BACKGROUND

In 1992, Mackowiak et al. suggested a revised definition of fever temperatures that attempts to correct for effects of the circadian cycle.[10] The proposal changes the threshold for fever to >37.2°C (>98.9°F) for oral temperatures taken in the morning and >37.7°C (>99.9°F) for those taken in the evening.[i] It is recommended in *Harrison's Internal Medicine*,[9] *UpToDate*,[11] and other prominent medical references, but does not appear to have been tested in any study since Mackowiak et al.'s initial report. Therefore, we decided that it would be valuable to examine this revised definition in the Boston and national emergency department data.

METHODS

Mackowiak et al. proposed a morning fever threshold of >37.2°C (>98.9°F), which was derived from a set of body temperatures measurements taken at 6 AM, as well as an evening fever threshold of >37.7°C (>99.9°F), which was derived from a set of body temperatures measurements taken at 4 PM. We applied Mackowiak et al.'s fever thresholds to Boston and NHAMCS body temperatures recorded at 6 AM and 4 PM. For other times of day, however, it was necessary to interpolate between Mackowiak et al.'s 6 AM and 4 PM thresholds in order to obtain reasonable definitions of fever. To do so, we used a Clausen function, which given by

$$f(x) = a + b \cdot \sum_{k=1}^{\infty} \frac{\sin(k \cdot x)}{k^z},$$

with $a = 99.4$, $b = 1.0052$, $z = 2.9$, and $x = \pi/12 \cdot (t - 11)$, where t is time of day in hours. With these parameters, the Clausen function matches Mackowiak et al.'s fever thresholds exactly at the specified times of 6 AM and 4 PM, and also varies smoothly between these thresholds at other times.

We chose a Clausen function for the following reason: While a simple sine curve may appear to be the most natural choice, in sine curves the minimum and maximum value are required to be exactly half a wavelength apart (i.e., 12 hours apart for a 24-hour cycle). The Clausen function is a visually similar alternative in which the minimum and maximum can be closer together, thereby allowing the minimum and maximum temperatures to be only 10 hours apart (6 AM and 4 PM), as specified in Mackowiak et al.'s fever definition.

---

[i] At some locations in Mackowiak and colleague's report, the definition is modified from > to ≥ 37.2°C in the morning and from > to ≥ 37.7°C in the evening. We applied the ">" definition because it is more common.

The data sources and other statistical methods are as explained in the main text.

Results and Discussion

Mackowiak et al.'s revised fever definition appeared to overcorrect in both the Boston and adult nationally representative data, resulting in a reversed pattern of fever incidence that was more than 2-fold higher in the early morning than in the afternoon (Appendix 8: Supplementary Figure 9). The overcorrection was observed both at the times for which Mackowiak et al. reported specific fever thresholds (6 AM and 4 PM) and at all other times of day, for which we used an interpolation procedure to obtain fever thresholds (*Methods*). Moreover, when using Mackowiak et al.'s revised fever definition, the overall incidence of fever was more than twice as high than observed when using the common fever definition of ≥38.0°C (≥100.4°F) (Boston data: 7.4% vs. 2.9%, national data: 6.7% vs. 3.0%).

The reasons for the overcorrections are unclear, but may be attributable to the small sample size of morning temperatures in Mackowiak et al.'s original study. In particular, Mackowiak et al. based their morning definition of the fever range on 19 temperature measurements taken from a group of healthy adults at 6 AM.[10(Fig. 2)] They calculated a 99th percentile from these measurements and used it as the morning fever threshold. This raises two issues: First, since the 99th percentile is defined as the value that is higher than 99% of all measurements, it is not possible to calculate a 99th percentile from only 19 measurements. Second, a much larger sample size is needed to obtain repeatable results from a statistical perspective. In short, if Mackowiak et al.'s procedures had been applied to a larger sampler size, very different fever thresholds could have been obtained. Although we view Mackowiak et al.'s study as valuable and groundbreaking research for its time, according to today's stronger standards of medical evidence, the small morning sample size is not sufficient to determine a diagnostic threshold for use in clinical practice.

On the other hand, another possible reason for the overcorrection is the use of different types of thermometry used in our data sources (Boston: temporal artery thermometry; national: a nationally representative sample of thermometer types used at emergency departments, consisting of a mixture of oral, temporal, tympanic, rectal, and other modalities) and by Mackowiak et al. (oral thermometry). Because different thermometer types were used, we cannot exclude the possibility that Mackowiak et al.'s fever thresholds are appropriate for oral thermometers, but not for the mixture of thermometer types used in our data sources.

To our knowledge, our evaluation is the first test of Mackowiak et al.'s revised definition of fever. Currently, there is no appropriate method to correct definitions of fever for the circadian cycle and additional research is needed to allow better corrections.

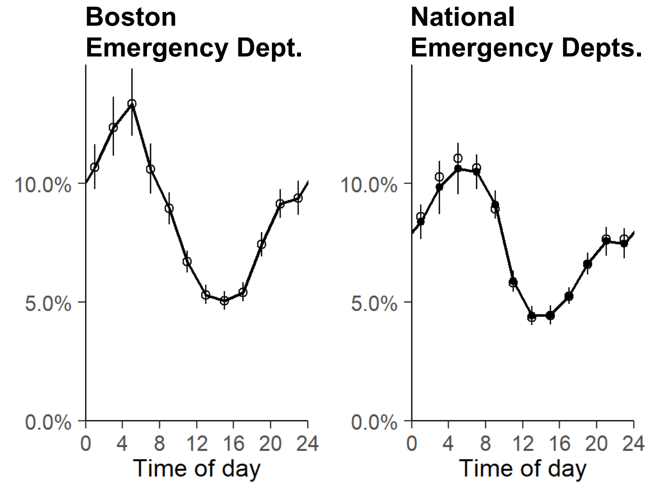

Supplementary Figure 9. Incidence of fever recorded at triage, using Mackowiak et al.'s method of defining fever.

This definition uses a fever threshold that varies by time of day, and was proposed to correct for circadian variation in healthy body temperature. However, the method appears to overcorrect, leading to a higher incidence of fever being recorded in the early morning, and comparatively depressed fever incidence being recorded in the afternoon. (The recorded incidence of fever is defined as the proportion of patents who present to emergency departments with a triage temperature that meets the definition of fever.) Confidence intervals are 95%. For the national data, the solid points show results obtained using multivariable regression to control for the 12 potential confounders, while the hollow points show the uncontrolled results. (The solid and hollow points are often so similar that they are difficult to distinguish.) National data are for ages ≥18 and Boston data come from an emergency department that mainly treats adults.

References

1. Graubard BI and Korn EL. Predictive margins with survey data. *Biometrics*. 1999;55(2):652-659.

2. Bieler GS, Brown GG, Williams RL, et al. Estimating model-adjusted risks, risk differences, and risk ratios from complex survey data. *Am J Epidemiol*. 2010;171(5):618-623.

3. Lumley T. survey: Analysis of complex survey samples. R package version 3.31-5. 2016.

4. Lumley T. *Complex Surveys: A Guide to Analysis Using R*. Hoboken: John Wiley & Sons, Ltd.; 2010.

5. Barbi E, Marzuillo P, Neri E, et al. Fever in children: pearls and pitfalls. *Children*. 2017;4(9):81.

6. Mirmiran M, Maas YGH, and Ariagno RL. Development of fetal and neonatal sleep and circadian rhythms. *Sleep Med Rev*. 2003;7(4):321-334.

7. Lodemore M, Petersen SA, and Wailoo MP. Development of night time temperature rhythms over the first six months of life. *Arch Dis Child*. 1991;66:521-524.

8. Joseph D, Chong NW, Shanks ME, et al. Getting rhythm: how do babies do it? *Arch Dis Child Fetal Neonatal Ed*. 2015;100:F50-F54.

9. Dinarello C and Porat R. Fever and Hyperthermia. In: Kasper DL, Fauci AS, Hauser SL, et al., eds. *Harrison's Principles of Internal Medicine*. 18th ed. New York: McGraw-Hill Education Medical; 2015:122-126.

10. Mackowiak PA, Wasserman SS, and Levine MM. A critical appraisal of 98.6 degrees F, the upper limit of the normal body temperature, and other legacies of Carl Reinhold August Wunderlich. *JAMA*. 1992;268(12):1578-1580.

11. Porat R and Dinarello C. Pathophysiology and treatment of fever in adults. UpToDate.
